# Comparison of Sensory and Motor Innervation Between the Acupoints LR3 and LR8 in the Rat With Regional Anatomy and Neural Tract Tracing

**DOI:** 10.3389/fnint.2021.728747

**Published:** 2021-09-01

**Authors:** Dongsheng Xu, Ling Zou, Wenjie Zhang, Jieying Liao, Jia Wang, Jingjing Cui, Yuxin Su, Yuqing Wang, Yating Guo, Yi Shen, Wanzhu Bai

**Affiliations:** ^1^Institute of Acupuncture and Moxibustion, China Academy of Chinese Medical Sciences, Beijing, China; ^2^South China Research Center for Acupuncture and Moxibustion, Medical College of Acu-Moxi and Rehabilitation, Guangzhou University of Chinese Medicine, Guangzhou, China; ^3^School of Traditional Chinese Medicine, Beijing University of Chinese Medicine, Beijing, China

**Keywords:** acupoints, meridian, regional anatomy, neural tract tracing, innervation, nervous system

## Abstract

**Objective:**

This study aimed to investigate the sensory and motor innervation of “Taichong” (LR3) and “Ququan” (LR8) in the rat and provide an insight into the neural relationship between the different acupoints in the same meridian.

**Methods:**

The LR3 and LR8 were selected as the representative acupoints from the Liver Meridian and examined by using the techniques of regional anatomy and neural tract tracing in this study. For both acupoints, their local nerves were observed with regional anatomy, and their sensory and motor pathways were traced using neural tract tracing with single cholera toxin subunit B (CTB) and dual Alexa Fluor 594/488 conjugates with CTB (AF594/488-CTB).

**Results:**

Using the regional anatomy, the branches of the deep peroneal nerve and saphenous nerve were separately found under the LR3 and LR8. Using single CTB, the sensory neurons, transganglionic axon terminals, and motor neurons associated with both LR3 and LR8 were demonstrated on the dorsal root ganglia (DRG), spinal dorsal horn, Clarke’s nucleus, gracile nucleus, and spinal ventral horn corresponding to their own spinal segments and target regions, respectively. Using dual AF594/488-CTB tracing, it was shown that the sensory and motor neurons associated with LR3 were separated from that of LR8.

**Conclusion:**

This study demonstrates that LR3 and LR8 are innervated by different peripheral nerves, which originated from or terminated in their corresponding spinal segments and target regions independently through the sensory and motor pathways. These results provide an example for understanding the differential innervation between the different acupoints in the same meridian.

## Introduction

The two representative acupoints on the hind limbs namely “Taichong” (LR3) and “Ququan” (LR8) are attributed to the Liver Meridian of Foot-Jueyin in the traditional acupuncture theory (Cheng, 1987; Liang, 2005). Although both of the acupoints have been located more precisely at the level of the regional anatomy in modern acupuncture science, their innervation was mainly introduced from the perspective of the peripheral nervous system (PNS) and less involved in the central nervous system (CNS; White and Editorial Board of Acupuncture in Medicine, 2009; Langevin et al., 2010; Cheng, 2011; Dorsher, 2017).

In order to reveal the differential innervation between LR3 and LR8 from the PNS to the CNS, their neural characteristics were systematically examined by the techniques of regional anatomy and neural tract tracing on the experimental rats. The two techniques have been used popularly in acupuncture research (Cui et al., 2013a,b, 2019; Wu et al., 2015; Wang et al., 2018; Lee et al., 2019; Wang H. et al., 2019; Wang J. et al., 2019). Herein, regional anatomy was used to show the peripheral nerves under the regions of LR3 and LR8, and neural tract tracing was applied to trace the origin and termination of the sensory and motor innervation from both acupoints to the CNS. According to our previous studies, single cholera toxin subunit B (CTB) and dual Alexa Fluor 594/488 conjugated with CTB (AF594/488-CTB) were selected for neural tract tracing in this study (Wang et al., 2018; Cui et al., 2019; Wang H. et al., 2019; Zhang et al., 2021). With the peripheral application, the single CTB is capable of retrogradely and transganglionically labeling the sensory and motor neurons and the sensory axon terminals (Cui et al., 2013a, 2019; Wang et al., 2018). Additionally, dual AF594/488-CTB are preferentially used in pair to retrogradely trace the sensory and motor neurons for identifying the neuronal correlation between the different acupoints or between the acupoint and its corresponding visceral organ (Cui et al., 2013b; Wang H. et al., 2019; Zhang et al., 2021).

With both anatomical and histological approaches, this study focused the observation on the nerves under the regions of LR3 and LR8, and their neural components at the cellular level along the sensory and motor pathways. Besides the traditional consideration of the LR3 and LR8 from the perspective of Meridians, this study is expected to provide the neuroanatomical evidence for explaining the differential innervation of different acupoints in the same meridian *via* the neural pathways.

## Materials and Methods

### Animals

This study used nine young adult male Sprague Dawley rats [8–10 weeks old, weight 200–230 g, license no. SCXK (JING) 2019-0010]. All the animals were housed in standard cages on a natural light-dark cycle with controlled temperature and humidity, and free access to food and water. The care and experimental manipulation of rats were approved by the Ethics Committee at the Institute of Acupuncture and Moxibustion, China Academy of Chinese Medical Sciences (reference number 2021-04-15-1).

### Microinjection of CTB Into the LR3 and LR8 on the Opposite Side of Hindlimb

Six rats were used for the injection of CTB into the LR3 and LR8 on the opposite sides of hindlimbs. Considering the application of the CTB on the peripheral site only produced neural labeling on the same side of injection, bilateral injections were performed in this study. This is also in order to reduce the number of animals used. Under the anesthesia with 2% isoflurane, 2 μl of 1% CTB solution (List Biological Labs, Inc., Campbell, CA, United States) was injected subcutaneously and muscularly with Hamilton micro-syringe (Hamilton Company, Reno, NV, United States) into the right side of LR3 and the left side of LR8, respectively.

**FIGURE 1:**
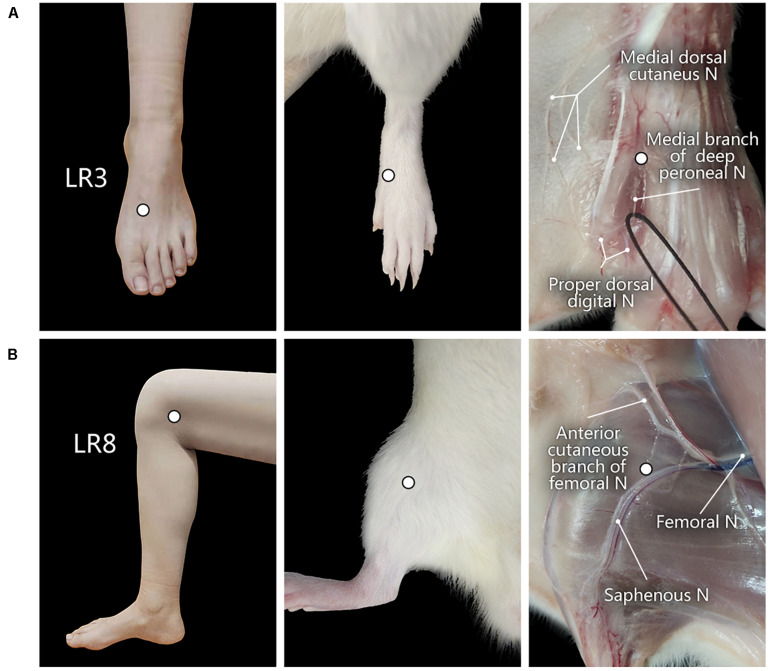
Location of acupoints “Taichong” (LR3) and “Ququan” (LR8) and their regional anatomy. **(A,B)** The sites of LR3 **(A)** and LR8 **(B)** in humans and rats, as well as their regional views of nerves (N) in rats.

The corresponding sites of LR3 and LR8 in the rat were determined according to the principle of comparative anatomy (Xu et al., 2019). For the human, LR3 is located on the dorsum of the foot, in the depression distal to the junction of the first and second metatarsal bones, while LR8 is located in the depression of the anterior border of the insertions of semimembranosus muscle and semitendinosus muscle, and when the knee is flexed, the point is at the medial end of the transverse popliteal crease (Huang and Huang, 2007; Lim, 2010; Figure 1).

### Microinjection of AF594/488-CTB Into the LR3 and LR8 on the Same Side of Hindlimb

Besides the application of single CTB on the opposite sides of LR3 and LR8, the injection of dual AF594-CTB/AF488-CTB was also carried out on the ipsilateral side of LR3 and LR8 with the other three rats. In this process, 2 μl of 0.1% AF594-CTB and 2 μl of 0.1% AF488-CTB (Invitrogen, Molecular Probes, Eugene, OR, United States) were separately injected into the LR3 and LR8 on the same hindlimb.

### Perfusion

Three days after injection, the rats were anesthetized with an overdose of tribromoethanol solution (250 mg/kg) to induce euthanasia and then transcardially perfused with 100 ml of 0.9% physiological saline followed by 250–300 ml 4% paraformaldehyde in 0.1 M phosphate buffer (PB, pH 7.4).

### Regional Anatomy

After perfusion, regional anatomy was performed along the hindlimb to observe the peripheral nerves on the regions of LR3 and LR8 (Figure 1). After observation, the brain, spinal cord, and dorsal root ganglia (DRG) were dissected out and cryoprotected in 25% sucrose in 0.1 M PB (pH 7.4) at 4°C for further histological examination.

### Slice and Fluorescent Immunohistochemistry

The transverse sections of the brain and spinal cord and sagittal sections of DRG were cut at a thickness of 40 μm on a freezing microtome (Microm International GmbH HM 430, Thermo Fisher Scientific, Germany). Afterward, these were orderly collected in 0.1 M PB (pH 7.4) with the 12-hole Petri dish.

The neural labeling with CTB was stained by immunofluorescence. In brief, the slices were incubated in a blocking solution containing 3% normal donkey serum and 0.5% Triton X-100 (Biotopped, China) in 0.1 M PB for 30 min, then removed to goat anti-CTB (1:1000; List Biological Labs, Inc.) in 0.1 M PB containing 1% normal donkey serum and 0.5% Triton X-100 at 4°C for one night. On the next day, the slices were washed thoroughly with 0.1 M PB and exposed to donkey anti-goat Alexa Fluor 488 secondary antibody (Molecular Probes, Eugene, OR, United States) (1:500; Molecular Probes) for 1 h. After that, the slices were washed again and then mounted on slides (Superfrost) (Thermo, MicromInternational FSE, Germany). The slides were cover slipped with 50% glycerin before the observation.

It was noteworthy that the neural labeling with AF594/488-CTB can be directly observed without further staining.

### Observation

Tissue samples were scanned with a panoramic tissue slice scanner (VS120; Olympus, Japan). Accordingly, the slices with CTB labeling were further observed and recorded using a fluorescent microscope equipped with a digital camera (DP73, Olympus, Japan) or a laser scanning confocal microscope (FV1200, Olympus, Japan). All images were processed using Adobe Photoshop/Illustration CC2017 (Adobe Systems, San Jose, CA, United States). The anatomical structure of tissue slices from the brain stem and spinal cord was determined according to *The Rat Brain in Stereotaxic Coordinates* (Paxinos and Watson, 2006), and the segments of DRG were marked by the spinal vertebra.

### Statistical Analysis

The number of the labeled neurons was obtained from the six rats with the injection of CTB, in which sensory neurons were counted in all the slices of each DRG, and the motor neurons were counted in every 12th slice of the spinal cord. All the data were expressed as mean ± *SE* and processed with the software GraphPad Prism 8.0.2 (La Jolla, CA, United States). Since there is no distinct boundary between the slices of the spinal cord, the number of labeled motor neurons was counted together without further distinguishing their spinal segments.

## Results

### Regional Observation of the LR3 and LR8

With regional anatomy, the peripheral nerves associated with LR3 and LR8 were clearly observed under the naked eyes. The common peroneal nerve branches from the sciatic nerve run down along the leg and further gives out the branch of the deep peroneal nerve toward the region of LR3. The saphenous nerve branches from the femoral nerve run into the region of LR8 (Figure 1).

### Neural Labeling Associated With LR3 and LR8 by CTB

**FIGURE 2:**
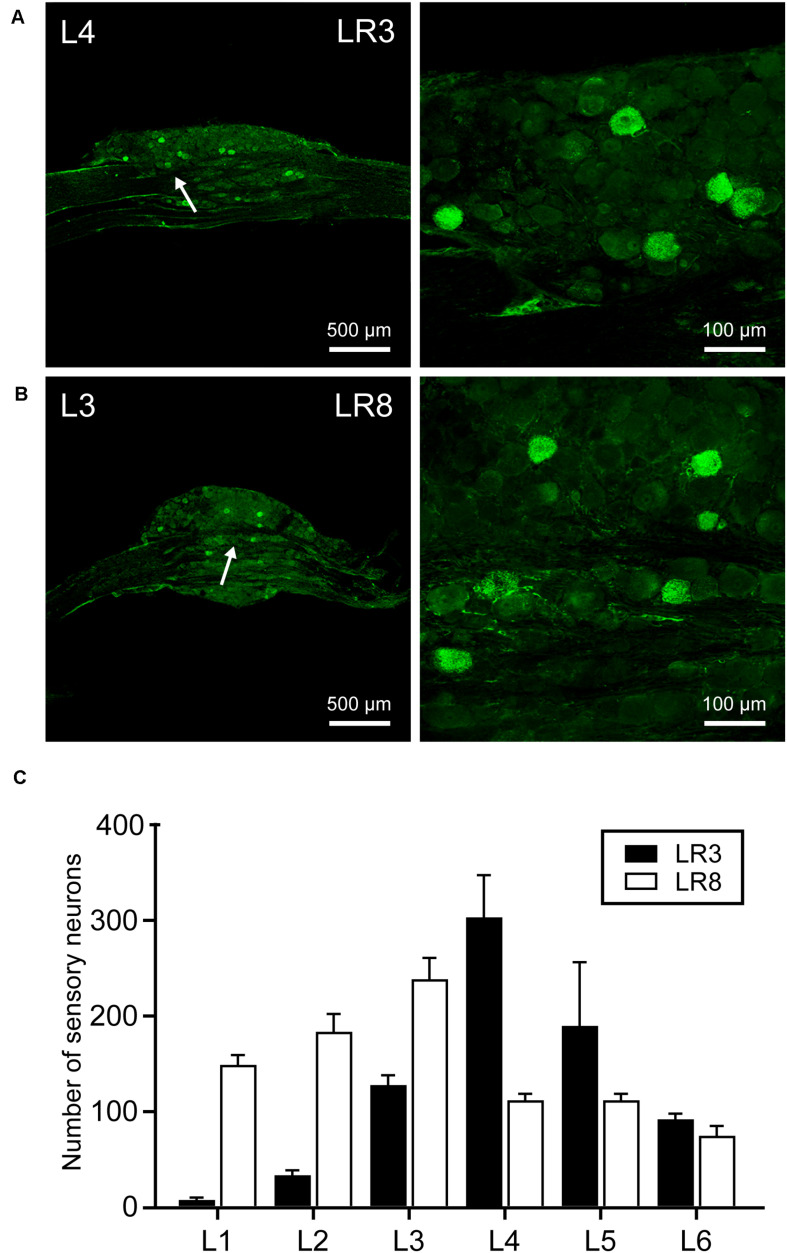
Distribution of the labeled sensory neurons with cholera toxin subunit B (CTB). **(A,B)** Representative photos from lumbar (L) 3 and L4 dorsal root ganglia (DRG) showing the CTB labeled sensory neurons associated with “Taichong” (LR3, **A**) and “Ququan” (LR8, **B**). And their magnified photos (arrowheads) showing the labeling in detail. **(C)** The distribution of CTB labeled sensory neurons for LR3 and LR8 from L1 DRG to L6 DRG in average number (*n* = 6).

**FIGURE 3:**
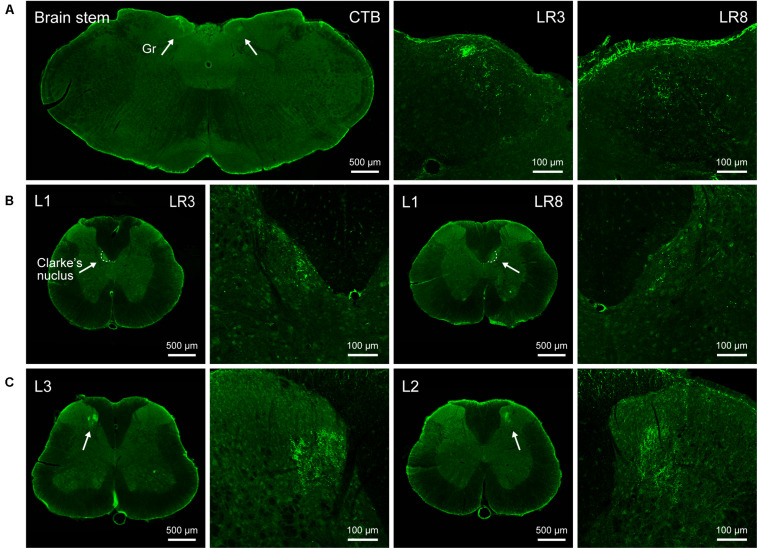
Distribution of the transganglionically labeled axon terminals with cholera toxin subunit B (CTB). **(A–C)** Representative photos from the brain stem **(A)** and spinal cord **(B,C)** showing the CTB labeled axon terminals associated with “Taichong” (LR3, left side) and “Ququan” (LR8, right side) in the gracile nucleus (Gr, **A**), Clarke’s nucleus at lumbar (L) 1 segment **(B)**, and spinal dorsal horns at L2 and L3 segments **(C)**, their magnified photos (arrowheads) showing the axon terminals in detail.

**FIGURE 4:**
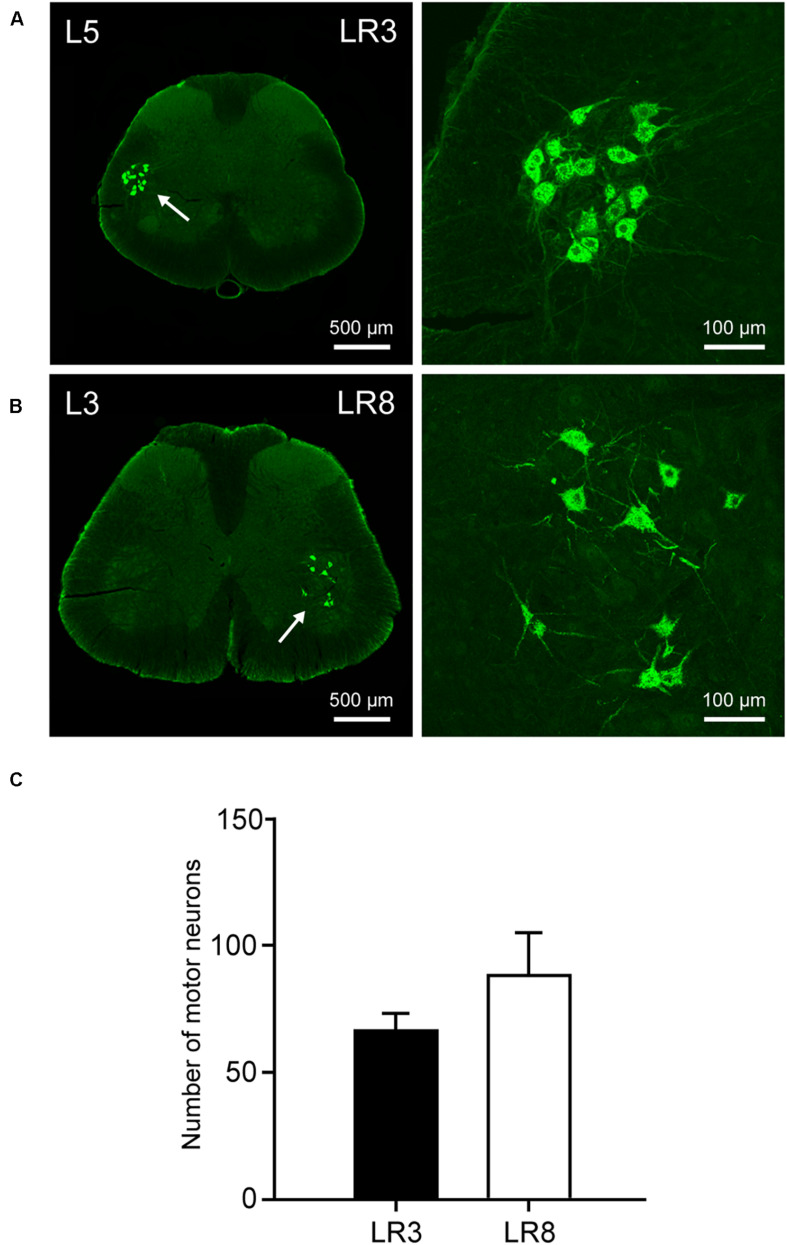
Distribution of the labeled motor neurons with cholera toxin subunit B (CTB). **(A,B)** Representative photos from lumbar (L) 5 and L3 spinal segments showing the CTB labeled motor neurons associated with “Taichong” (LR3, **A**) and “Ququan” (LR8, **B**), their magnified photos (arrowheads) showing the motor neurons in detail. **(C)** The average number of CTB labeled motor neurons for LR3 and LR8 (*n* = 6).

The neural components associated with LR3 and LR8 were demonstrated with CTB labeling, including the sensory neurons in DRG, transganglionic axon terminals in the spinal dorsal horn, Clarke’s nucleus, and gracile nucleus, as well as motor neurons in the spinal ventral horn. This labeling is located ipsilaterally corresponding to the side of CTB injection for LR3 and LR8, respectively (Figures 2–4).

The sensory neurons for the LR3 were distributed in lumbar (L) 1–6 DRG with higher concentration in L4 DRG (Figure 2), and the transganglionic axons were terminated on the dorsal part of the gracile nucleus, Clarke’s nucleus, and medial part of the spinal dorsal horn, respectively (Figure 3). On the other hand, the motor neurons were present on the lateral part of spinal ventral horns ranging from L3 to L6 segments with a higher concentration on the L4–5 (Figure 4). In the cases of LR8, the sensory neurons were also distributed in L1–6 DRG, but with higher concentration in L3 DRG (Figure 2), the transganglionic axons terminated on the ventral part of the gracile nucleus, Clarke’s nucleus, and central part of the spinal dorsal horn, respectively (Figure 3), while the motor neurons were presented on the ventral part of spinal ventral horns in the L2–5 with a higher concentration on the L3–4 (Figure 4).

### Neural Labeling Associated With LR3 and LR8 by AF594/488-CTB

**FIGURE 5:**
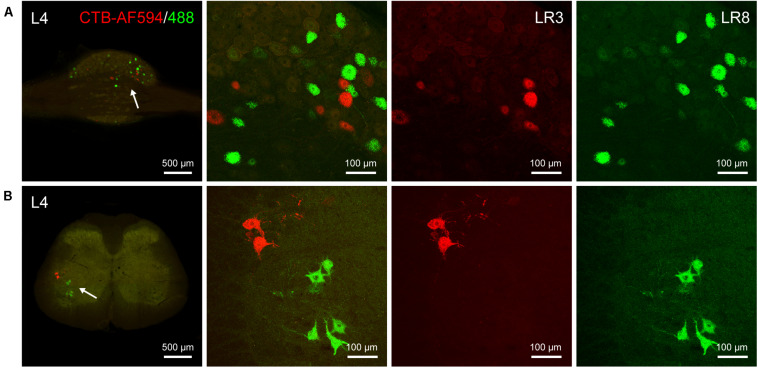
Distribution of the labeled sensory and motor neurons with Alexa Fluor 594 and 488 conjugated cholera toxin subunit B (AF594/488-CTB) in the dorsal root ganglion and spinal cord. **(A,B)** Representative photos from lumbar (L) 4 dorsal root ganglion **(A)** and spinal cord **(B)** showing the labeled sensory and motor neurons associated with “Taichong” (LR3, AF594-CTB, red) and “Ququan” (LR8, AF488-CTB, green), their magnified photos (arrowheads) showing the labeled sensory **(A)** and motor **(B)** neurons in detail.

The differential innervation between LR3 and LR8 was further compared with a dual fluorescent tracing technique (Figure 5). Unlike CTB, only sensory and motor neurons associated with LR3 and LR8 were retrogradely labeled with AF594/488-CTB, but the distribution of these labeled neurons was similar to that of CTB. Although some of the sensory and motor neurons associated with LR3 and LR8 intermingled in the certain DRG and spinal ventral horn, these labeling was of separate neurons (Figure 5).

## Discussion

**FIGURE 6:**
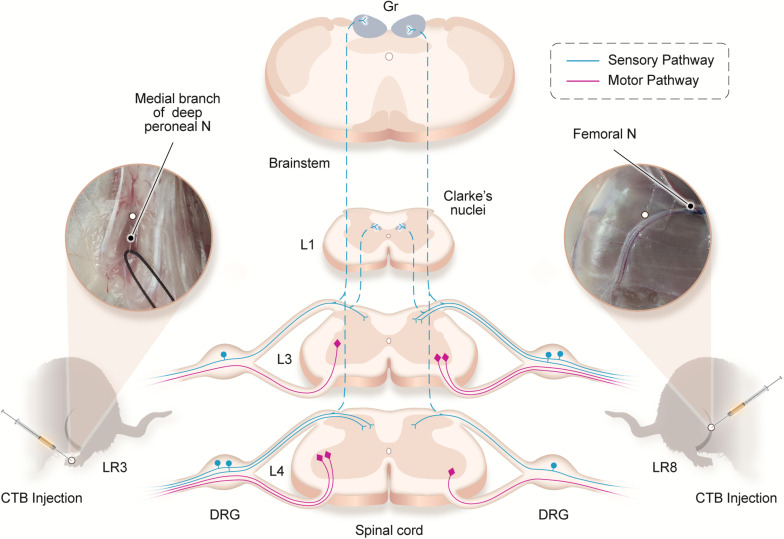
Illustration of the distribution of the neural labeling associated with “Taichong” (LR3) and “Ququan” (LR8) through the sensory and motor pathways. Acupoints LR3 and LR8 establish their sensory and motor connections with the corresponding targets in the dorsal root ganglion (DRG), spinal dorsal horns, Clarke’s nucleus, gracile nucleus (Gr), and spinal ventral horns in the segmental and regional pattern.

Traditionally, the meridians were considered as the pathways serving for the circulation of the qi and blood on the human body. They pertain to the zang-fu organs (visceral organs) interiorly and extend over the body exteriorly, forming a network and linking the tissues and organs into an organic whole. This system included the 12 main meridians running longitudinally and interiorly within the body, with each meridian considered to interconnect with its corresponding visceral organ. However, it is absent until now of structural basis to support this discussed interconnection. In our previous study, we have demonstrated the correlated sensory and sympathetic innervations between acupoint BL 23 and kidney in the rat (Zhang et al., 2021). In this study, we focused our observation on the differential innervation between the different acupoints. We demonstrated a detailed view of the neuroanatomical characteristics of LR3 and LR8 with the techniques of regional anatomy and neural tract tracing in the rats. Besides the consideration from the traditional theory of meridians, present observation provides new insight into the neuroanatomical difference between the LR3 and LR8 *via* the sensory and motor pathways. From the PNS to the CNS, it is beneficial for understanding the effective routes of the two acupoints by acupuncture stimulation (Figure 6).

### Neuroanatomical Consideration

The peripheral nerves under the LR3 and LR8 in the rats were demonstrated by the regional anatomy in this study. Similar to the two acupoints on the human body, they are innervated by the peroneal nerve and saphenous nerve, respectively (Cheng, 1987; Liang, 2005; Huang and Huang, 2007). This peripheral difference is further determined with neural tract tracing between the LR3 and LR8 at the cellular level. The sensory neurons, transganglionic axon terminals, and motor neurons associated with LR3 and LR8 are distributed orderly corresponding to their own spinal segments and target regions along the sensory and motor pathways from the PNS to the CNS.

Although the neural components are the same for the innervation of both LR3 and LR8, their segmental and regional distribution is markedly different between each other. For the spinal segments, the concentration of sensory and motor labeling associated with LR3 is obviously one segment lower than that of LR8. For the target regions, it is mainly exhibited on the distribution of the transganglionic axon terminals and motor neurons. The transganglionic axon terminals associated with LR3 locate more medially in the spinal dorsal horn than that of LR8, and the motor neurons associated with LR3 are situated more laterally on the spinal ventral horn than that of LR8. These segmental and regional distribution is accorded with the “somatotopic arrangement” in the CNS of rats, which has been well defined in the previous studies (Swett and Woolf, 1985; Maslany et al., 1991, 1992; Takahashi et al., 2010; Odagaki et al., 2019). Increasing pieces of evidence from the human have proved that “somatotopic arrangement” does not only exist on the cerebral cortices but is also present in the spinal cord (Standring, 2008; Stroman et al., 2012; Willoughby et al., 2021).

According to the distribution of the transganglionically labeled axons in the CNS, our results imply that the acupuncture signals from the LR3 and LR8 can be transported to their corresponding targets in the spinal dorsal horn, Clarke’s nucleus, and gracile nucleus, and forwarded *via* these central relay stations to the contralateral side of ventral posterolateral thalamic nucleus and the ipsilateral cerebellum through the spinothalamic, medial lemniscus, and spinocerebellar tracts, respectively (Standring, 2008). Comparatively, the motor innervation of LR3 and LR8 is uncomplicated, originating from the motor neurons in the spinal ventral horn, where the motor neurons associated with LR3 locate more laterally than those of LR8. Taken together, it can be concluded that there is a segmental and regional difference of sensory and motor innervation between LR3 and LR8, which gives out a clue leading to understanding their effective routes of acupuncture stimulation from the perspective of sensory and motor pathways.

### Clinical Implication

Since acupoints are considered as the specific sites for acupuncture, how to select a proper acupoint becomes an important issue to be continuously concerned in the clinical application (Cheng, 1987; Huang and Huang, 2007; Chiang, 2015). Generally, selecting acupoints along the route of meridians is the basic principle in acupuncture treatment, which is carried out according to the theory that diseases are related to meridians (Liang, 2005; Liu et al., 2010). Despite both LR3 and LR8 belonging to the same meridian, this study suggests that their neuroanatomical specificity should be another important consideration when they are selected as the proper acupoints for acupuncture treatment (Dorsher, 2017; Guo and Ma, 2019).

From the viewpoint of neural pathways, present neuroanatomical findings from the regional anatomy and neural tract tracing suggest that although LR3 and LR8 are directly innervated by the peroneal and saphenous nerves, respectively, they may also involve in their successive parts of sciatic and femoral nerves, even in their corresponding spinal segments and target regions. Accordingly, the acupuncture signals aroused from LR3 and LR8 might be transported along these neural pathways to the CNS for the precise target treatment. Therefore, the results of this study provide valuable references for evaluating the therapeutic roles of LR3 and LR8.

### Study Limitations

The primary focus of this study was the identification and characterization of the differential innervation between the LR3 and LR8 at the anatomic and cellular levels. Although our study provides the neuroanatomical pieces of evidence that the sensory and motor pathways may participate in the processes of signal transportation from the LR3 and LR8 to the CNS, their functional effects remain to be established experimentally.

Due to the technical limitation, all the neural labeling with CTB and AF594/488-CTB along the sensory and motor pathways belongs to the first-order neural components associated with the LR3 and LR8. Their successive higher-order neural components remain unclear, which might be transsynaptically traced with neurotropic viruses in the future study (Lanciego and Wouterlood, 2011, 2020; Saleeba et al., 2019). In addition, it is worth noting that besides the nerve fibers, the other cellular components are also distributed in the tissues under the acupoints, which should be an important issue to be tackled in the future study (Zhang et al., 2012; Wu et al., 2015; Xu et al., 2016; She et al., 2018).

## Conclusion

This study evidently showed the neuroanatomical characteristics of LR3 and LR8. Although both acupoints are attributed to the same meridian, considering their sensory and motor innervation, each of them establishes its own neural connection with corresponding spinal segments and target regions from the PNS to the CNS *via* the sensory and motor pathways. These results suggest that when we select the proper acupoints for the acupuncture treatment, the neuroanatomical specificity of acupoints should also be taken together as the clinical demands, besides the consideration from the traditional theory of meridians.

## Data Availability Statement

The raw data supporting the conclusions of this article will be made available by the authors, without undue reservation.

## Ethics Statement

The animal study was reviewed and approved by the Ethics Committee of the Institute of Acupuncture and Moxibustion, China Academy of Chinese Medical Sciences.

## Author Contributions

The manuscript presented here was carried out in collaboration between all authors. DX, LZ, WZ, JL, YSu, YG, and YSh carried out the experiments. JW and JC analyzed the data. WB designed the study. DX and WB wrote the article. All authors read and approved the final version of the manuscript.

## Conflict of Interest

The authors declare that the research was conducted in the absence of any commercial or financial relationships that could be construed as a potential conflict of interest.

## Publisher’s Note

All claims expressed in this article are solely those of the authors and do not necessarily represent those of their affiliated organizations, or those of the publisher, the editors and the reviewers. Any product that may be evaluated in this article, or claim that may be made by its manufacturer, is not guaranteed or endorsed by the publisher.
